# Correction: Expert-augmented automated machine learning optimizes hemodynamic predictors of spinal cord injury outcome

**DOI:** 10.1371/journal.pone.0294081

**Published:** 2023-11-02

**Authors:** Austin Chou, Abel Torres-Espin, Nikos Kyritsis, J. Russell Huie, Sarah Khatry, Jeremy Funk, Jennifer Hay, Andrew Lofgreen, Rajiv Shah, Chandler McCann, Lisa U. Pascual, Edilberto Amorim, Philip R. Weinstein, Geoffrey T. Manley, Sanjay S. Dhall, Jonathan Z. Pan, Jacqueline C. Bresnahan, Michael S. Beattie, William D. Whetstone, Adam R. Ferguson, the TRACK-SCI Investigators

An additional affiliation is missing for author Adam R. Ferguson, who is also affiliated with San Francisco Veterans Affairs Healthcare System, San Francisco, California, United States of America.

The following information is missing from the Data Availability statement: Source data has been deposited to the Open Data Commons for Spinal Cord Injury (odc-sci.org;RRID:SCR_016673) under the accession number ODC-SCI:727 (http://doi.org/10.34945/F5KG6Z).
