# Supplementary figures and images for: Expert-augmented automated machine learning optimizes hemodynamic predictors of spinal cord injury outcome
Source: PLoS One. 2022 Apr 7;17(4):e0265254. doi: 10.1371/journal.pone.0265254 (PMC8989303; doi:10.1371/journal.pone.0265254)

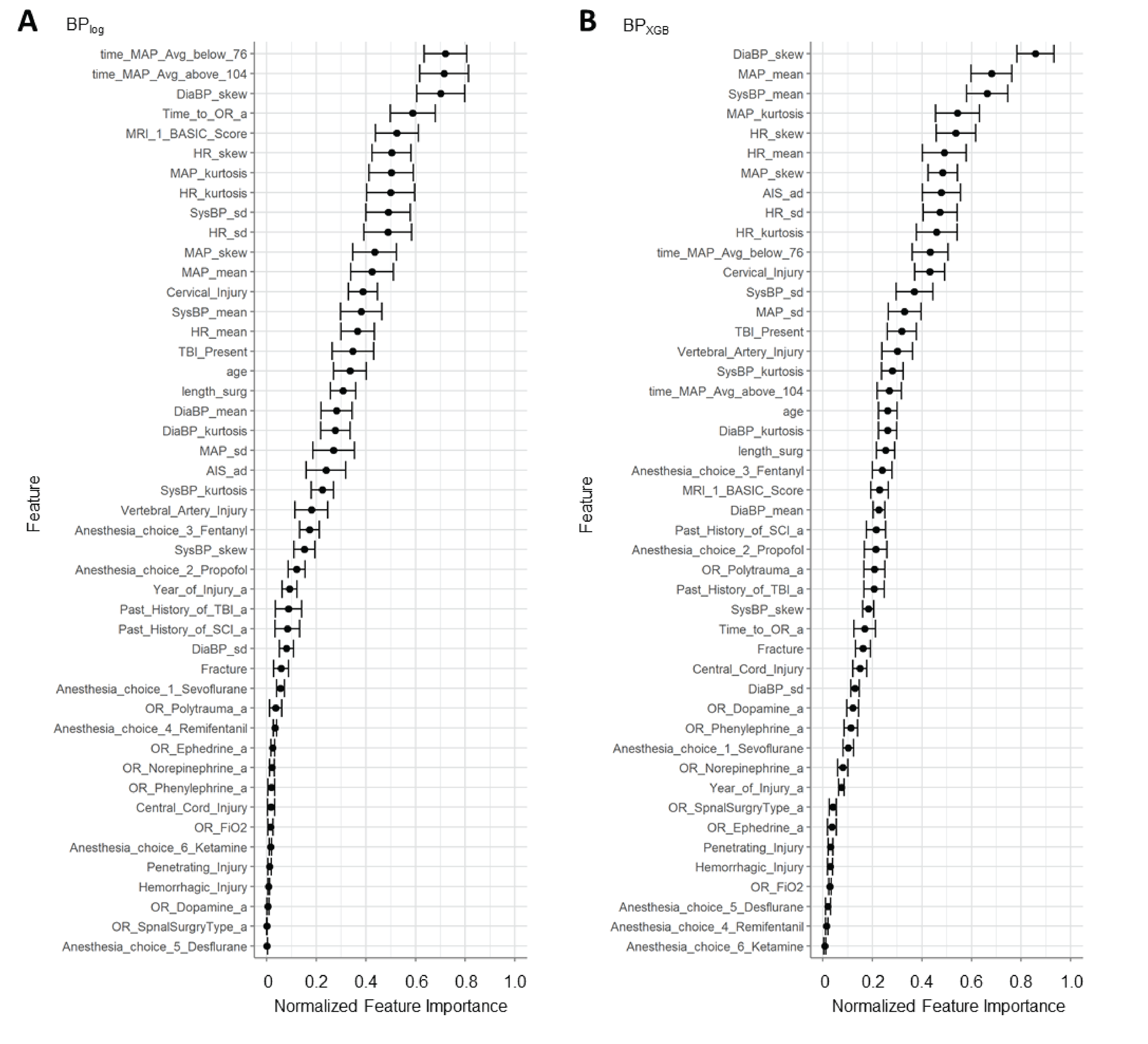

Supplement: S1 Fig — (A) Of note, BPlog ranked the time_MAP_Avg_below_76 and time_MAP_Avg_above_104 highest. (B) Conversely, the two MAP threshold-related features were ranked 11th and 18th in pFI by BPXGB. The majority of high pFI features across both models were features derived from the intraoperative timeseries data for heart rate, diastolic blood pressure, systolic blood pressure, and mean arterial pressure (MAP). Both models also highly ranked a feature encoding initial injury severity: MRI_1_BASIC_Score for BPlog and AIS_ad for BPXGB. (TIF) [file pone.0265254.s001.tif]

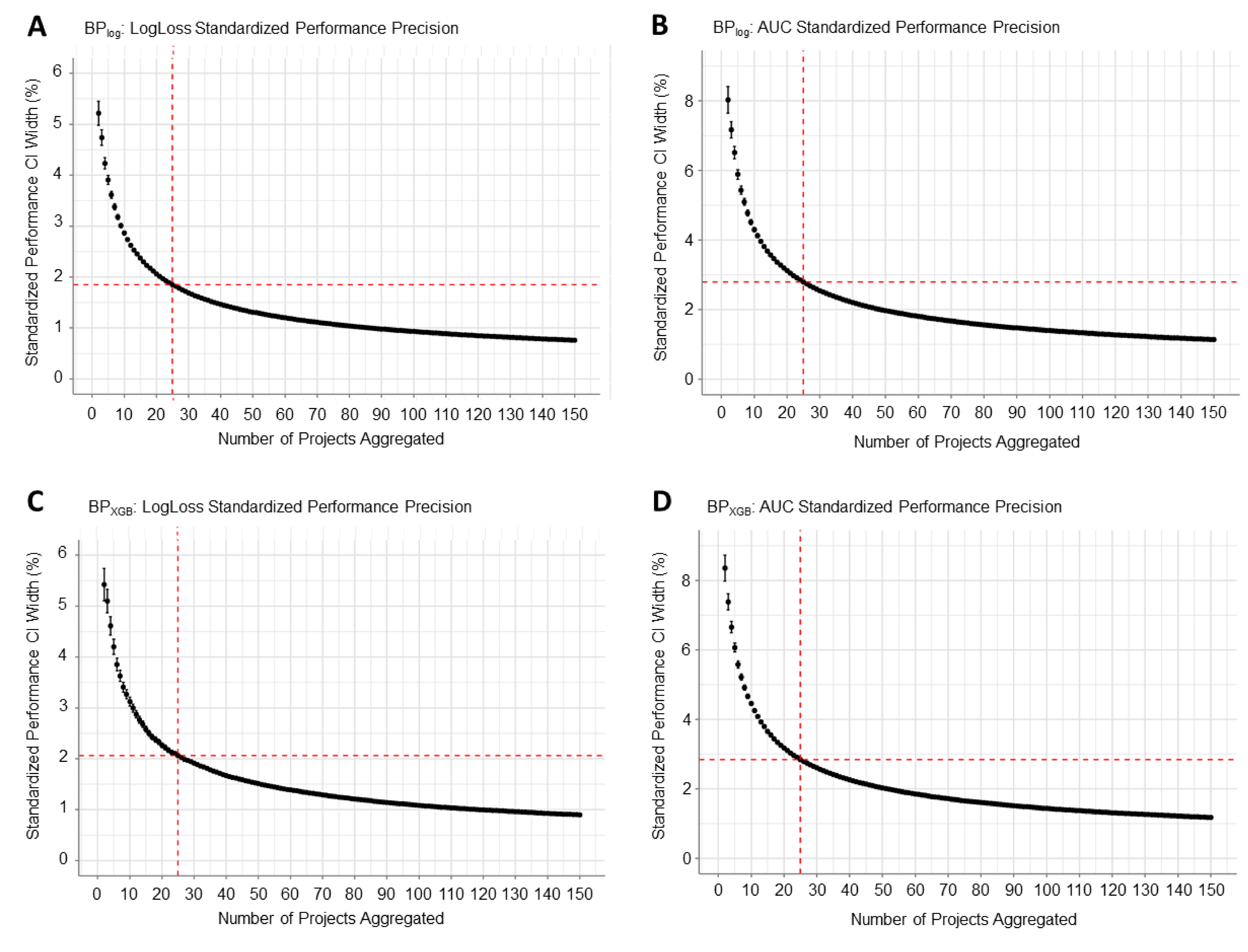

Supplement: S2 Fig — As the number of projects increased, the performance precision improved (i.e. standardized performance CI width decreased). (A, B) By LogLoss, BPlog started with a standardized performance precision of 5.22 ± 0.24% with 2-project aggregation and decreased to an average of 1.85 ± 0.01% with 25-project aggregation (A). By AUC, BPlog started with a performance precision of 8.03 ± 0.38% and decreased to an average of 2.79 ± 0.02% when aggregating 25 projects. (C, D) Similarly by LogLoss, BPXGB started with a standardized performance precision of 5.42 ± 0.32% and decreased to an average of 2.06 ± 0.04% at 25 projects (C). By AUC, BPXGB started with a performance precision of 8.36 ± 0.38% and decreased to an average of 2.84 ± 0.02% when aggregating 25 projects. (TIF) [file pone.0265254.s002.tif]

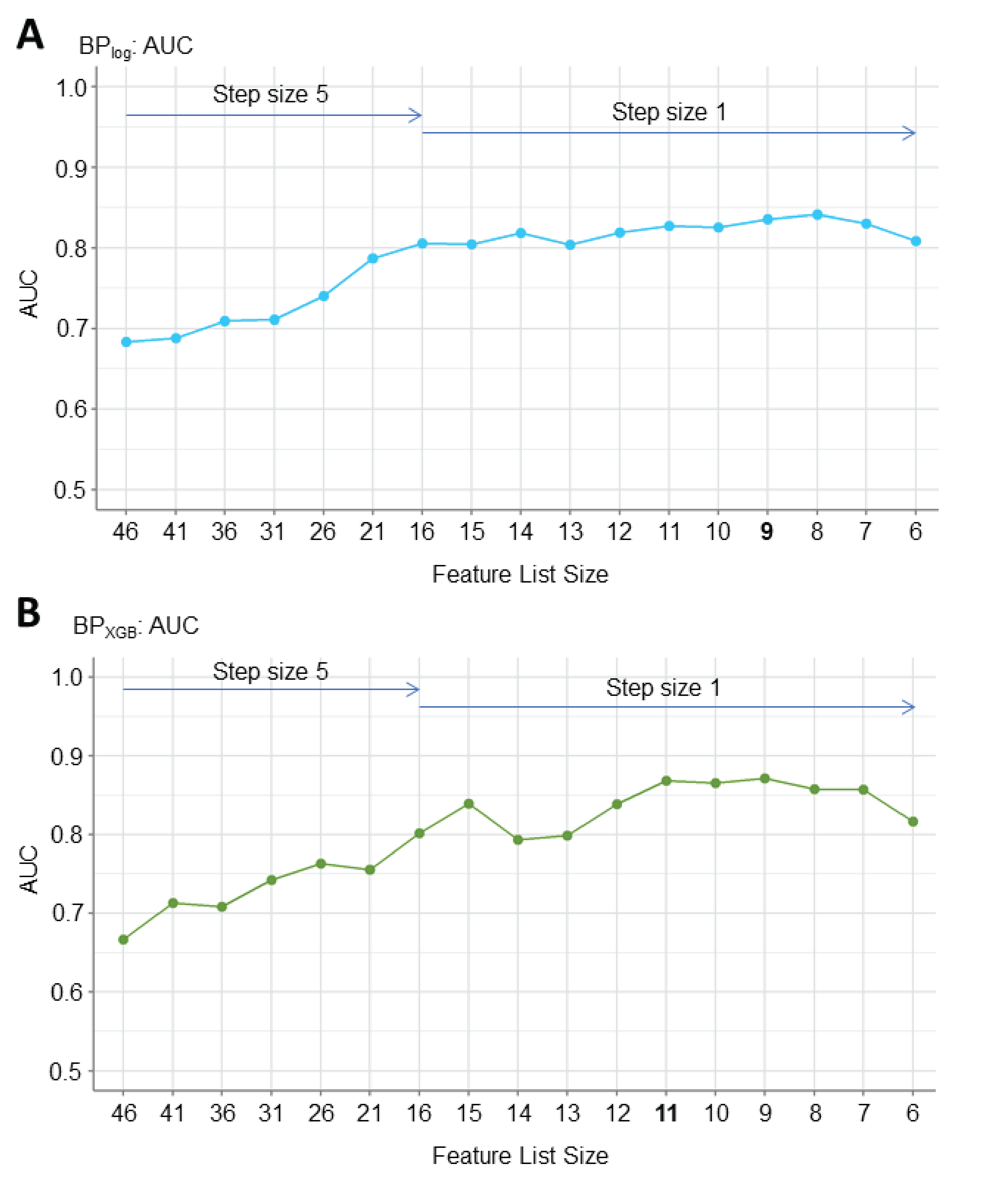

Supplement: S3 Fig — (A) Feature reduction of BPlog showed maximum AUC at the 8-feature parsimonious feature list (AUC = 0.84 ± 0.02). The 9-feature parsimonious feature list had an AUC of 0.83 ± 0.02. (B) Feature reduction of BPXGB showed maximum AUC at the 9-feature parsimonious feature list (AUC = 0.87 ± 0.01). The 11-feature parsimonious feature list had a similar AUC of 0.87 ± 0.01. (TIF) [file pone.0265254.s003.tif]

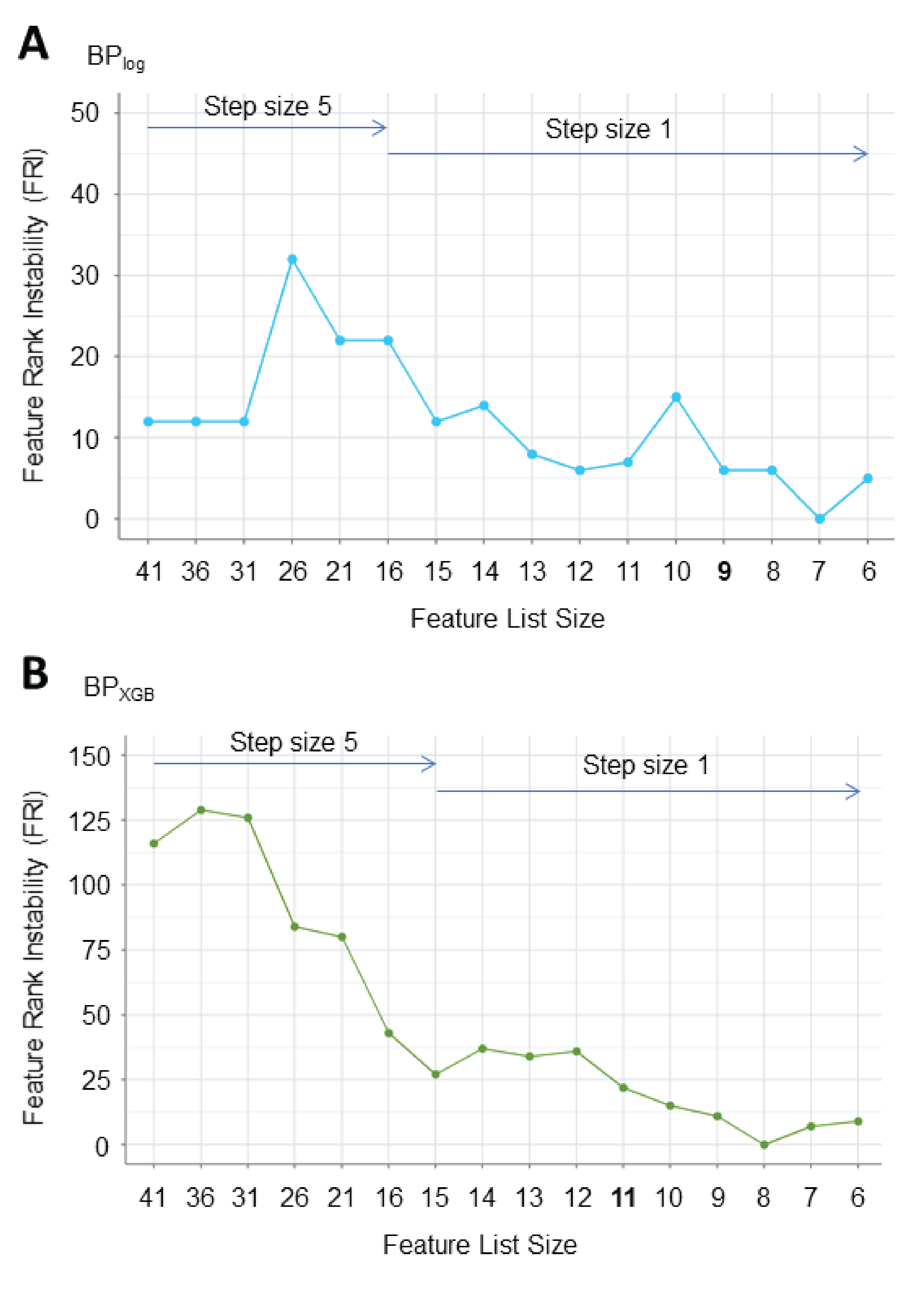

Supplement: S4 Fig — FRI was calculated by comparing the pFI ranking before and after each feature reduction step and only summing the features that appeared in both lists (i.e. features that were not removed at the step). Notably, BPXGB exhibited higher FRI at each step than for BPlog; elimination of features resulted in more shifting of features by pFI rank for BPXGB. (TIF) [file pone.0265254.s004.tif]

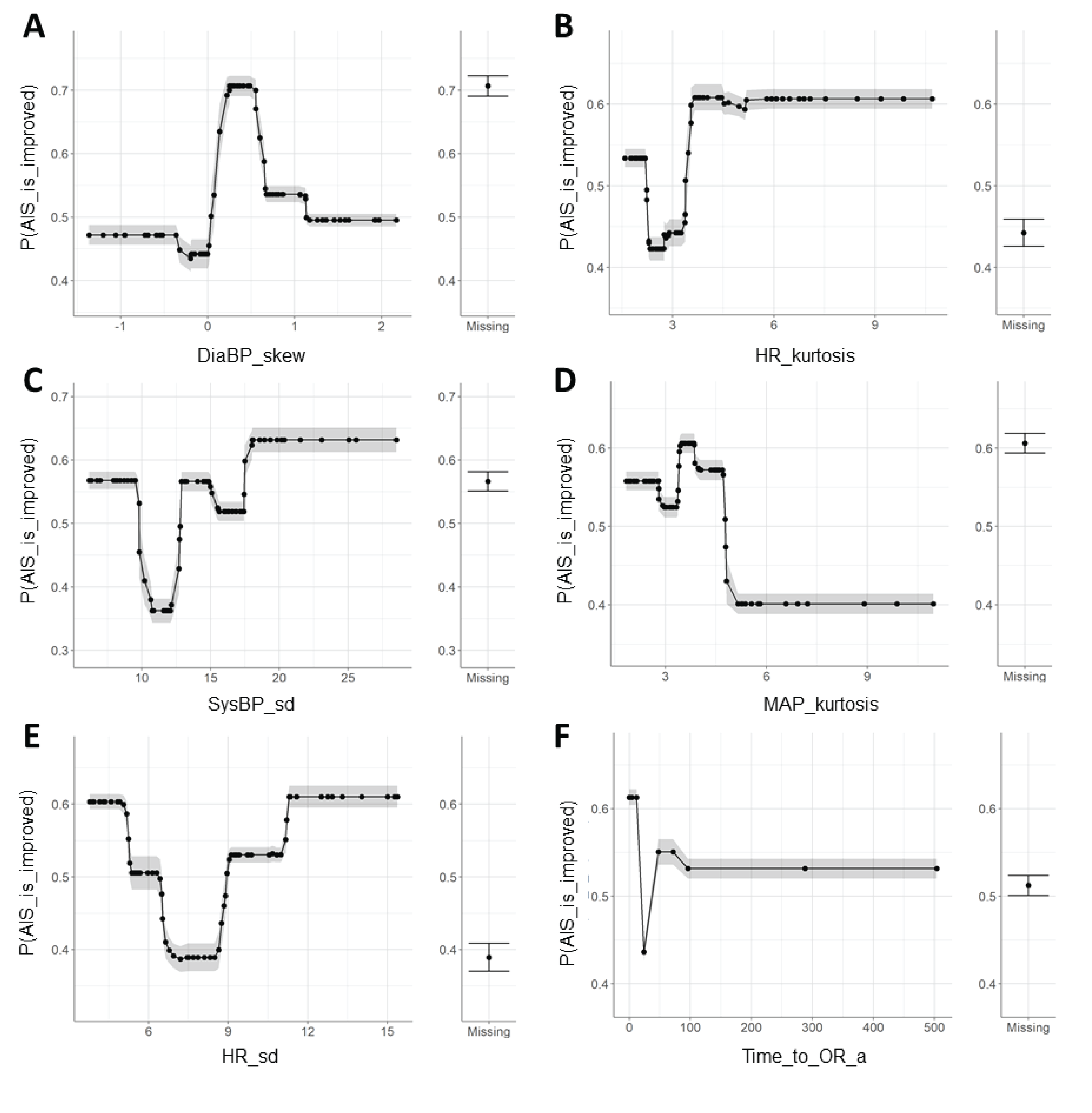

Supplement: S5 Fig — In order of highest pFI to lowest: (A) DiaBP_skew, (B) HR_kurtosis, (C) SysBP_sd, (D) MAP_kurtosis, (E) HR_sd, and (F) Time_to_OR_a. PDPs of MRI_1_BASIC_Score, time_MAP_Avg_above_104, and time_MAP_Avg_below_76 are shown in Fig 4. (TIF) [file pone.0265254.s005.tif]

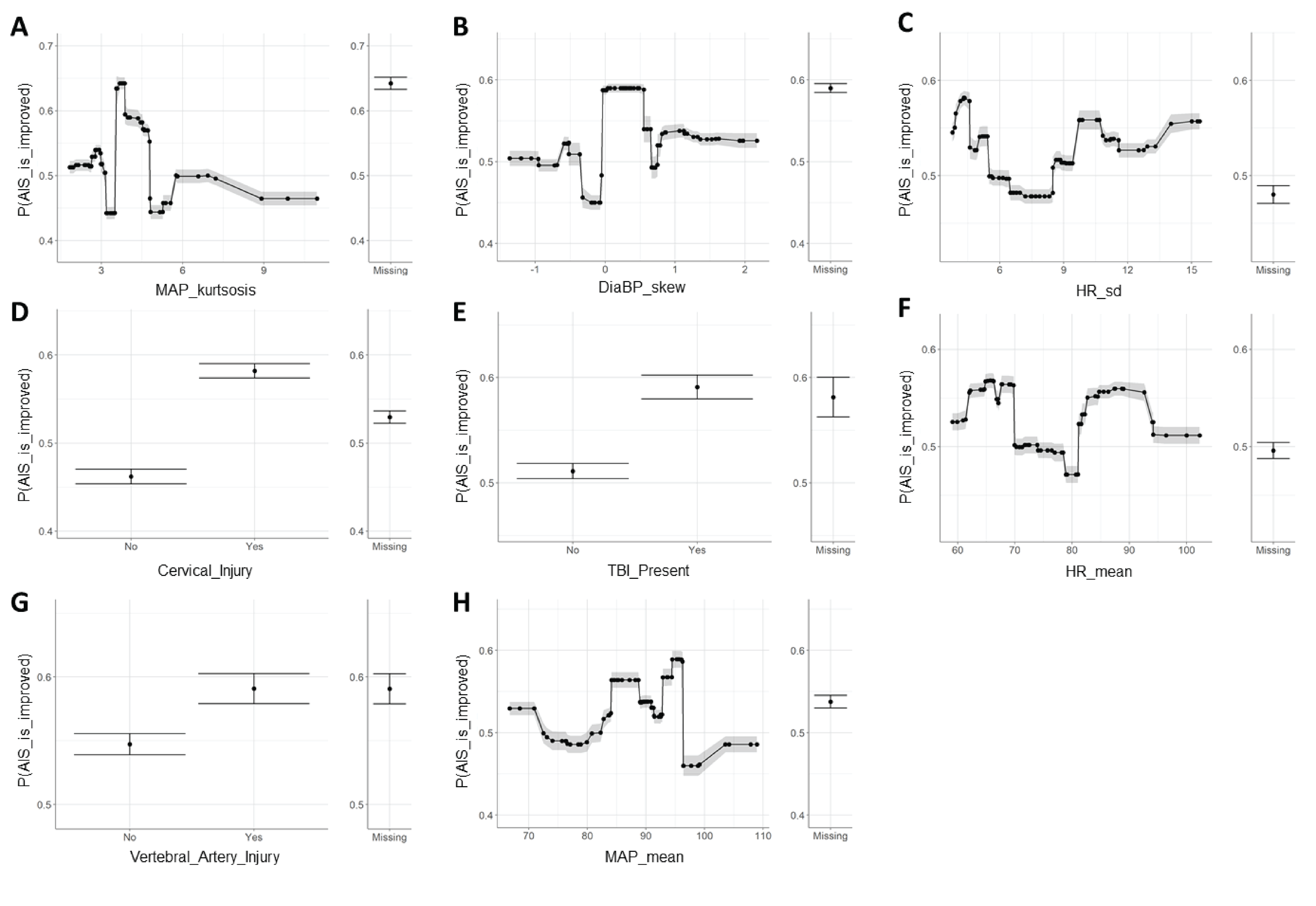

Supplement: S6 Fig — In order of highest pFI to lowest: (A) MAP_kurtosis, (B) DiaBP_skew, (C) HR_sd, (D) Cervical_Injury, (E) TBI_Present, (F) HR_mean, (G) Vertebral_Artery_Injury, and (H) MAP_mean. PDPs of AIS_ad, time_MAP_Avg_above_104, and time_MAP_Avg_below_76 are shown in Fig 4. (TIF) [file pone.0265254.s006.tif]

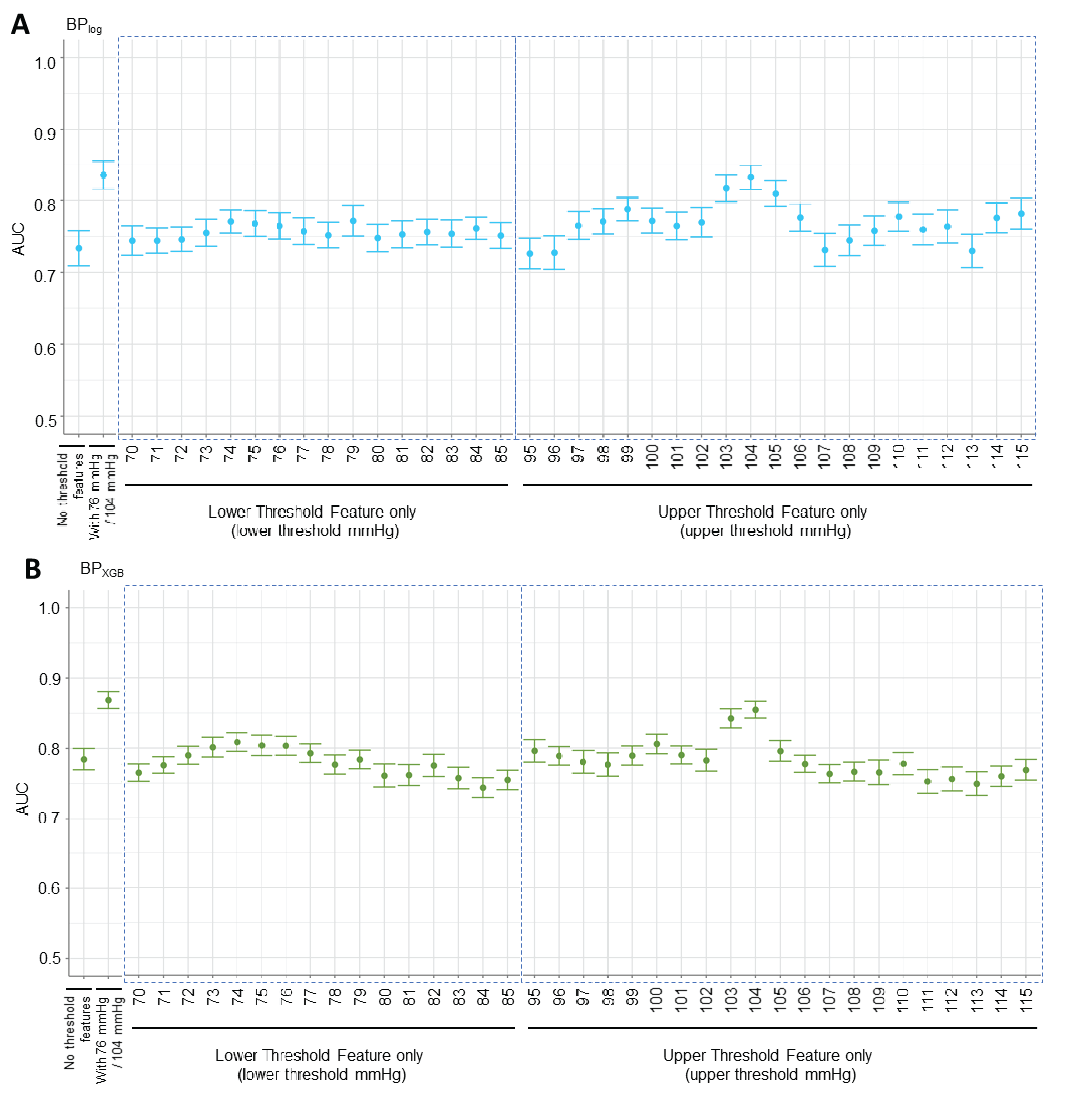

Supplement: S7 Fig — (A) Similar to the LogLoss plots, the best-performing lower threshold values were 74, 75, 76, and 79 mmHg and the best-performing upper threshold values were 103, 104, and 105 mmHg for BPlog. Of the best-performing thresholds, inclusion of an upper threshold features produced greater improvement to AUC than inclusion of an individual lower threshold feature. (B) For BPXGB, the best-performing lower threshold values were 74, 75, and 76 mmHg, and the best-performing upper threshold values were 103 and 104 mmHg. Similar to BPlog, of the best-performing thresholds, inclusion of an individual upper threshold feature improved AUC performance more than inclusion of an individual lower threshold feature. (TIF) [file pone.0265254.s007.tif]

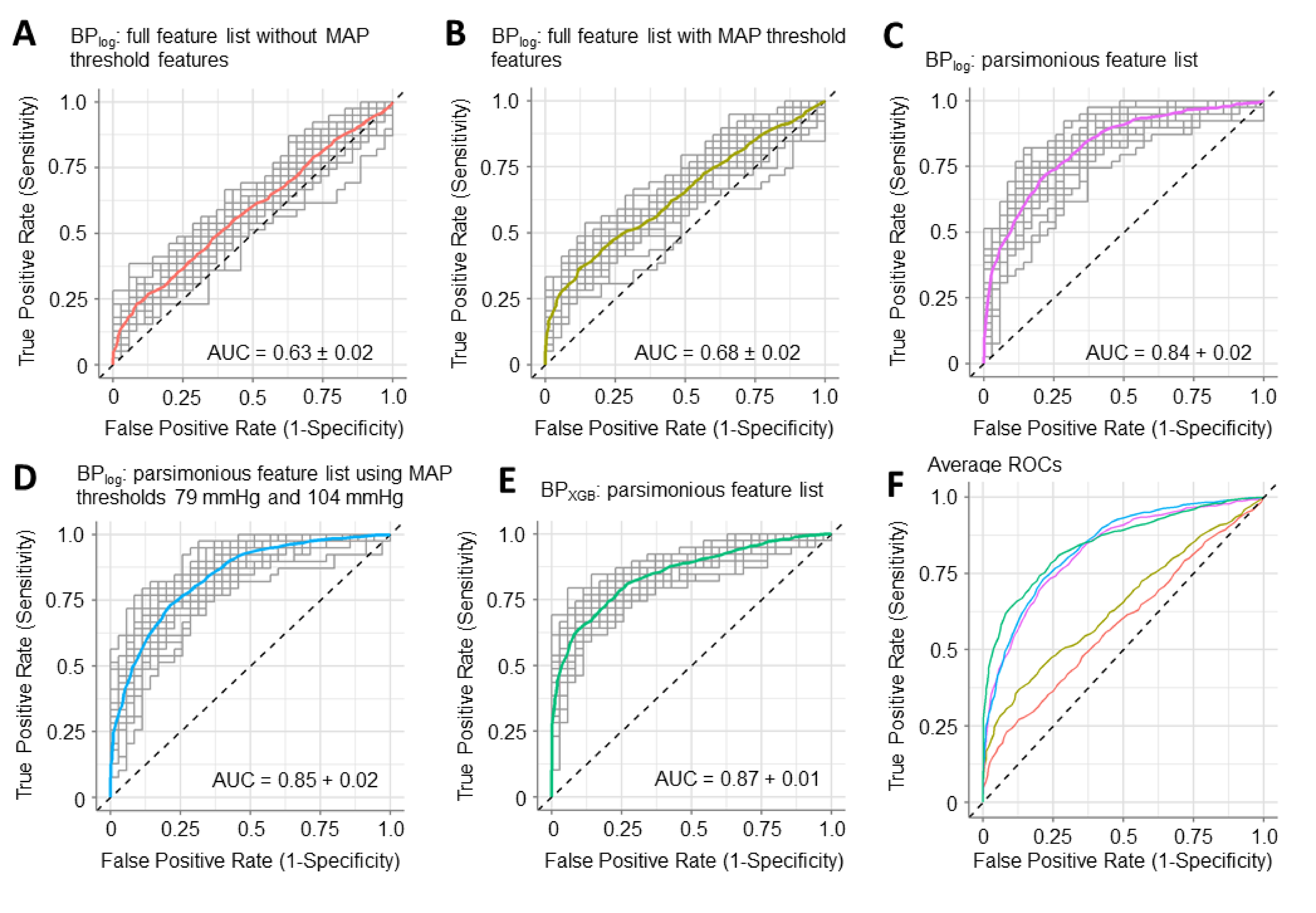

Supplement: S8 Fig — (A) ROC curves of the L2 regularized linear regression model BPlog trained on the initial feature list with the exclusion of the MAP threshold features. The average model AUC was 0.63 ± 0.02. (B) ROC curves of BPlog trained on the full feature list including the two MAP threshold features. The average AUC was 0.68 ± 0.02. (C) ROC curves after performing feature reduction with BPlog to find the best-performing parsimonious model (9-feature parsimonious feature list). The average AUC increased to 0.84 ± 0.02. (D) ROC curves after testing different MAP thresholds with BPlog and selecting for the best-performing lower (79 mmHg) and upper (104 mmHg) thresholds. The resulting AUC improved incrementally (AUC 0.85 ± 0.02) compared to using 76 mmHg and 104 mmHg. (E) ROC curves after performing the workflow on the eXtreme gradient boosted tree model BPXGB. The parsimonious feature list consisted of 11 features and the best-performing MAP thresholds were 76 and 104 mmHg. The average model AUC was 0.87 ± 0.01. (TIF) [file pone.0265254.s008.tif]

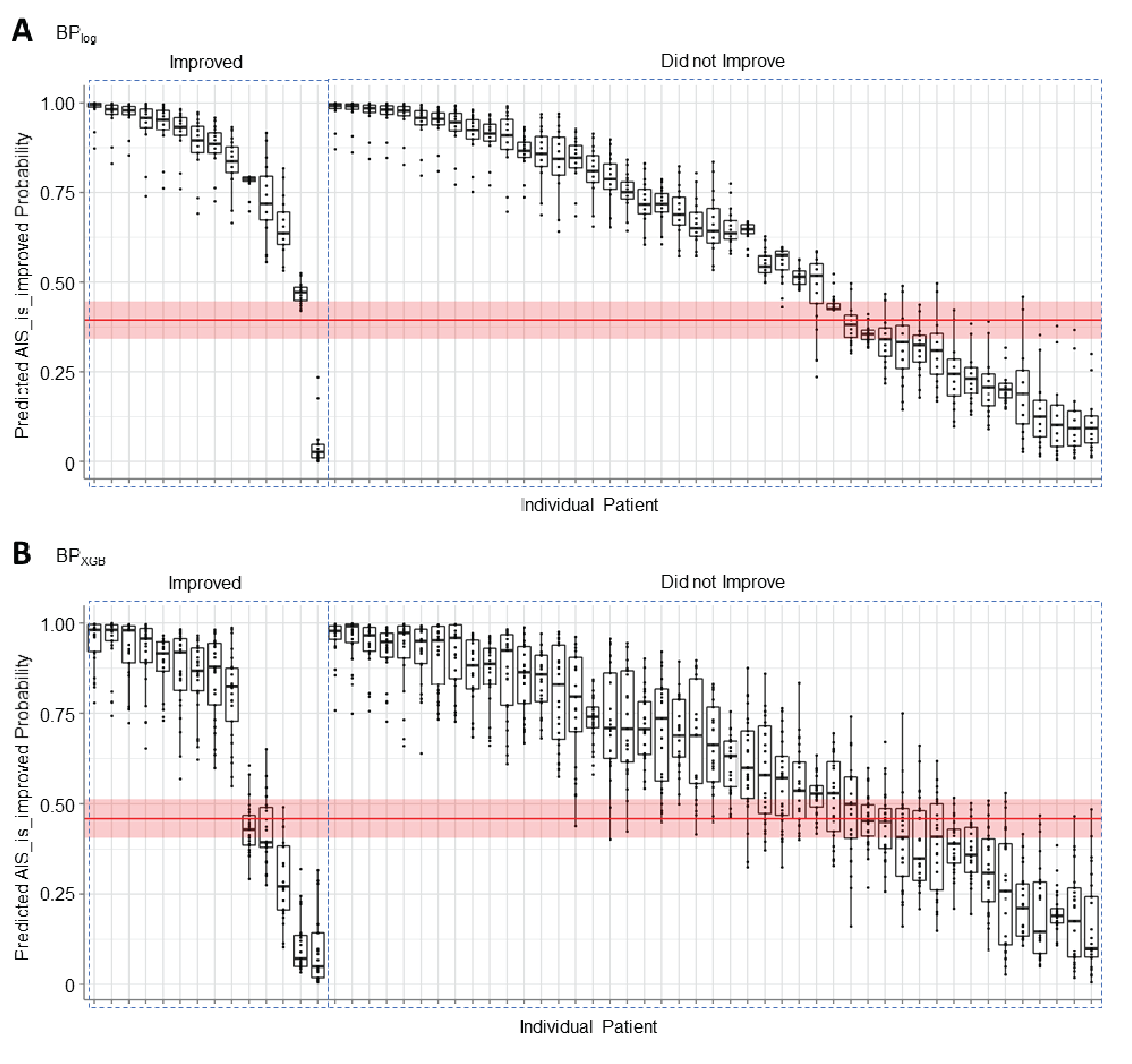

Supplement: S9 Fig — Of these, 14 patients improved in AIS score while 45 patients did not. Best F1 thresholds as calculated by the AutoML platform were also aggregated from each project (shown in red). (A) Prediction for each validation subject by BPlog. The average best F1 threshold is 0.41 ± 0.04. (B) Prediction for each validation subject by BPXGB. The average best F1 threshold is 0.46 ± 0.04. (TIF) [file pone.0265254.s009.tif]

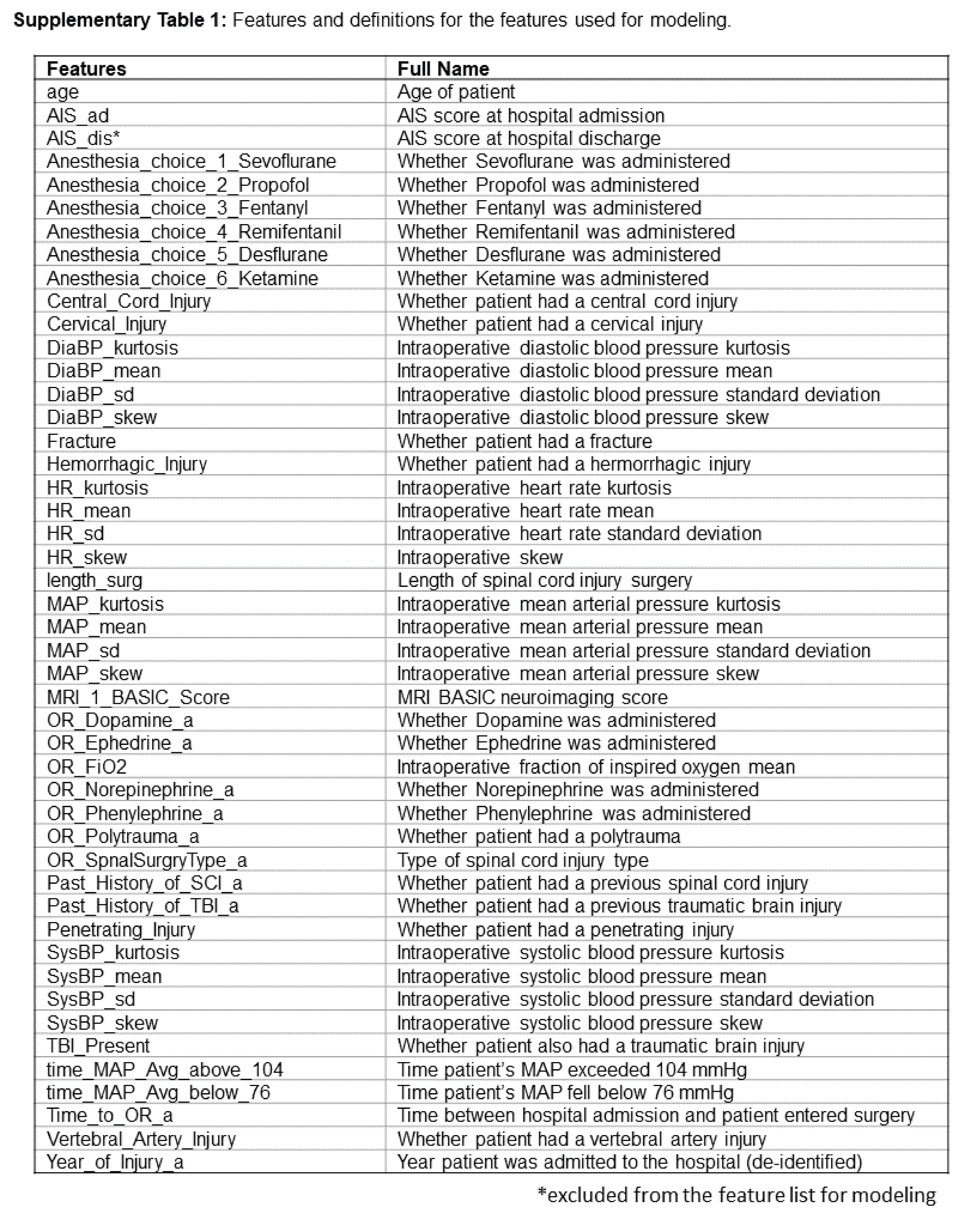

Supplement: S1 Table — (TIF) [file pone.0265254.s010.tif]

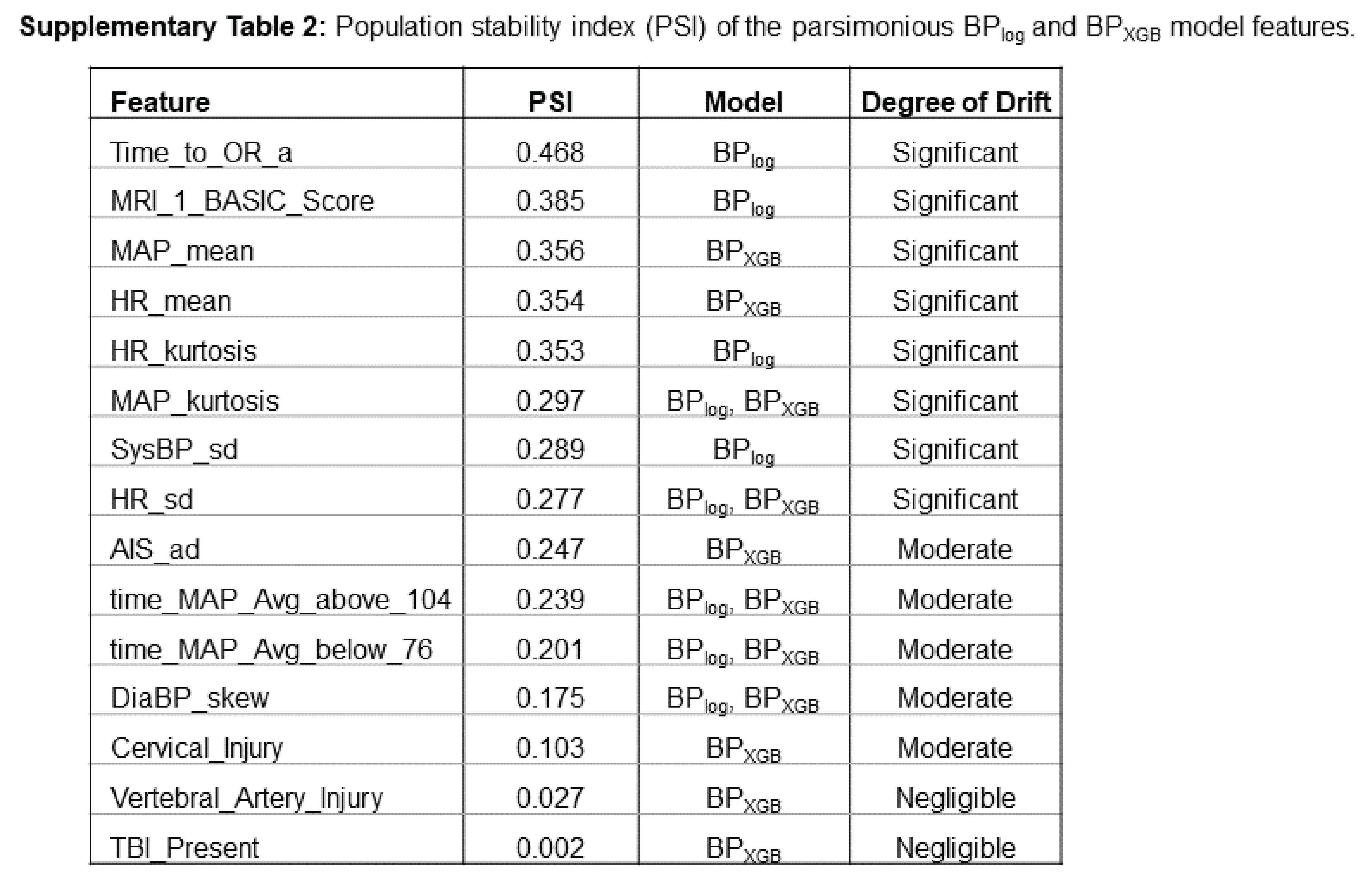

Supplement: S2 Table — (TIF) [file pone.0265254.s011.tif]

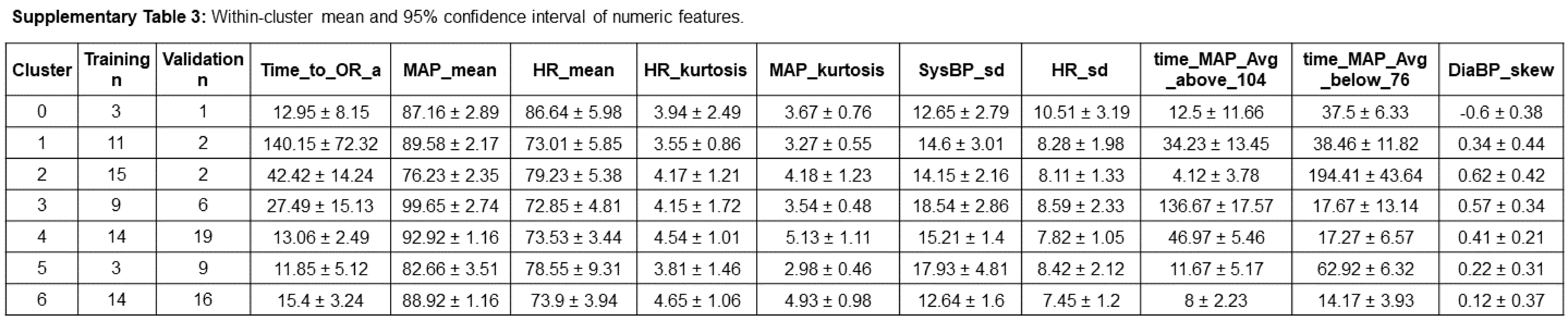

Supplement: S3 Table — (TIF) [file pone.0265254.s012.tif]
